# The Post-Synaptic Function of Brca2

**DOI:** 10.1038/s41598-019-41054-y

**Published:** 2019-03-14

**Authors:** Charles X. Wang, Judit Jimenez-Sainz, Ryan B. Jensen, Alexander V. Mazin

**Affiliations:** 1 Department of Biochemistry and Molecular Biology, Drexel University College of Medicine, Philadelphia, PA 19102 USA; 2 MD/PhD Program, Drexel University College of Medicine, Philadelphia, PA 19102 USA; 3 Department of Therapeutic Radiology, Yale University School of Medicine, New Haven, CT 06520 USA; 4 Present Address: Department of Radiation Oncology, MD Anderson Cancer Center, Houston, TX 77030 USA

## Abstract

Homologous Recombination (HR) is a high-fidelity process with a range of biologic functions from generation of genetic diversity to repair of DNA double-strand breaks (DSBs). In mammalian cells, BRCA2 facilitates the polymerization of RAD51 onto ssDNA to form a presynaptic nucleoprotein filament. This filament can then strand invade a homologous dsDNA to form the displacement loop (D-loop) structure leading to the eventual DSB repair. Here, we have found that RAD51 in stoichiometric excess over ssDNA can cause D-loop disassembly *in vitro*; furthermore, we show that this RAD51 activity is countered by BRCA2. These results demonstrate that BRCA2 may have a previously unexpected activity: regulation of HR at a post-synaptic stage by modulating RAD51-mediated D-loop dissociation. Our *in vitro* results suggest a mechanistic underpinning of homeostasis between RAD51 and BRCA2, which is an important factor of HR in mammalian cells.

## Introduction

Double-strand breaks (DSBs) represent the most lethal type of DNA damage occurring in the cell approximately 50 times per cell cycle^1^. If unrepaired or repaired erroneously, DSBs may cause cell death or genome rearrangements leading to tumorigenesis^2^. Homologous Recombination (HR) is the pathway that repairs DSBs with high fidelity^3^. In human cells, HR primarily proceeds with RAD51 protein binding to the ssDNA tails generated by resection of DSB ends to form a nucleoprotein filament. BRCA2, assisted by DSS1, facilitates this process by helping RAD51 to overcome the competition from Replication Protein A (RPA), a ubiquitous ssDNA binding protein^4–6^. Furthermore, BRCA2 helps to stabilize the RAD51-ssDNA filament by inhibiting the RAD51 ATPase activity^4,5^. These functions of BRCA2 depend on the BRC repeats, eight conserved motifs of approximately 35 amino acid residues each, that are responsible for interaction with RAD51^7^. Of the eight BRC repeats, BRC4 has the greatest affinity to RAD51^8^; individual BRC4 polypeptide can stimulate RAD51-ssDNA filament formation *in vitro*^4^.

During synaptsis, the RAD51-ssDNA filament searches for a homologous sequence in dsDNA and invades it to form the D-loop structure^9^. The homologous DNA within D-loops serves as a template for DNA polymerase to extend the invading ssDNA. After polymerase extension, the ssDNA dissociates from the D-loop and anneals to the second resected end of the DSB. This D-loop dissociation represents an important step during HR, which prevents formation of crossovers in mitotically dividing cells or reduces their formation to the optimal level during meiosis^10,11^.

Several helicase-like proteins or topoisomerases, including RAD54, Top3-Rmi1, BLM, RTEL1, and Mph1 are capable of dissociating D-loops^11–15^. Here, we demonstrate that RAD51 itself can dissociate the D-loops *in vitro*. Furthermore, we show that BRCA2 can control this dissociation. Previously, it has been shown that the homeostasis between RAD51 and BRCA2 plays an important role in HR in human cells^16,17^, however until now this observation lacked a clear molecular underpinning. Our current results may provide a molecular mechanism for this homeostasis. Maintaining the optimal RAD51/BRCA2 balance may be important for the formation of the active RAD51-ssDNA filament during presynaptic stage and for the proper timing of D-loop dissociation at the post-synaptic stage of DNA strand exchange.

## Results

### RAD51 can promote D-loop dissociation

**Figure 1:**
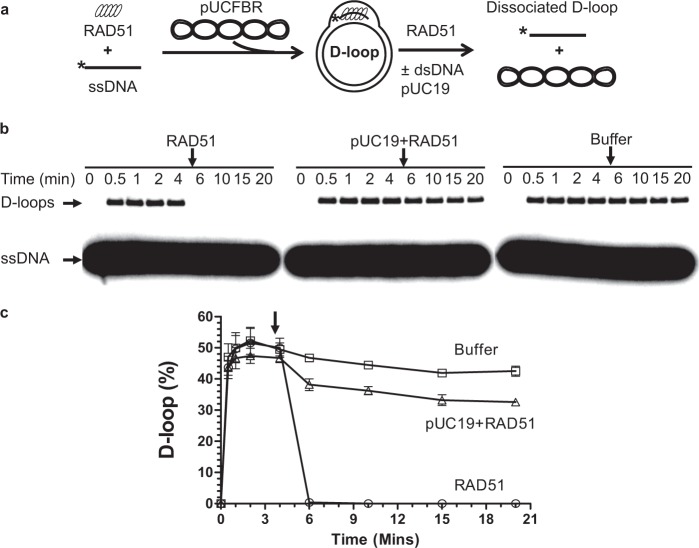
RAD51 promotes D-loop dissociation. (**a**) Scheme of D-loop dissociation by RAD51. The asterisk in this and following schemes indicates ^32^P-label on ssDNA. (**b**) Addition of RAD51 (1 µM) to the RAD51-generated D-loops on pUCFBR DNA (6.25 µM, nt) causes their dissociation. As controls, either RAD51 (1 µM) premixed with heterologous pUC19 scDNA (43.75 µM, nt) or RAD51 storage buffer were added to the reactions at the indicated time point. These and all subsequent D-loops were analyzed by autoradiography of 1% agarose gels following gel-electrophoresis and expressed as a percentage of the total homologous plasmid DNA (limiting factor). “0” time point on the graph corresponds to the initiation of D-loop formation. (**c**) The data from (**b**) represented as a graph. The vertical arrow marks addition of RAD51, RAD51 + pUC19, or buffer. Error bars indicate standard error of the mean (SEM); the experiments were repeated at least three times.

First, we initiated RAD51-promoted D-loop formation between ssDNA (oligo #160; 3 µM, nt; Table S1) and homologous plasmid pUCFBR scDNA (6.25 µM, nt). D-loop formation proceeded for 4 min to reach the maximal extent (~45–50%), then an additional amount of RAD51 was added to the ongoing reaction (Fig. 1a). We found that RAD51 (1 µM) does indeed dissociate its own D-loops (Fig. 1b-left panel,c). As a control, the addition of the RAD51 storage buffer did not have any effect on the D-loop yield indicating that the effect was intrinsic to RAD51 (Fig. 1b-right panel,c). D-loop dissociation by RAD51 was significantly reduced when RAD51 was pre-incubated with a scavenger heterologous pUC19 scDNA prior to addition to the D-loop reaction. This suggests that direct RAD51 binding to the D-loop is required for its dissociation (Fig. 1b-middle panel,c). A possibility that D-loop dissociation was caused by nicking of plasmid scDNA by contaminating nucleases was ruled out because no degradation or relaxation of superhelical plasmid DNA was seen in the agarose gels stained with ethidium bromide (EtBr) (Fig. S1). To evaluate the capability of RAD51 in dissociating D-loops, we deproteinized and purified RAD51-generated D-loops and subjected them to incubation with the varying quantities of RAD51 (Fig. S2a). We found that RAD51 dissociates D-loops in a concentration dependent manner, where 0.5 µM RAD51 was sufficient to achieve full dissociation of 25 µM (nt) of D-loops. (Fig. S2b,c). This RAD51 activity to dissociate the very D-loops that it produces may represent an important self-regulatory mechanism of HR.

### dsDNA unwinding by RAD51 is critical to D-loop dissociation

It is known that removal of negative superhelicity or generation of positive superhelicity in plasmid DNA destabilizes D-loops^18^. Therefore, we suggest that the ability of RAD51 to dissociate D-loops may be related to an intrinsic ability to unwind dsDNA; this DNA unwinding by RAD51 generates compensatory positive superhelical twists in plasmid DNA causing D-loop dissociation^19^.

**Figure 2:**
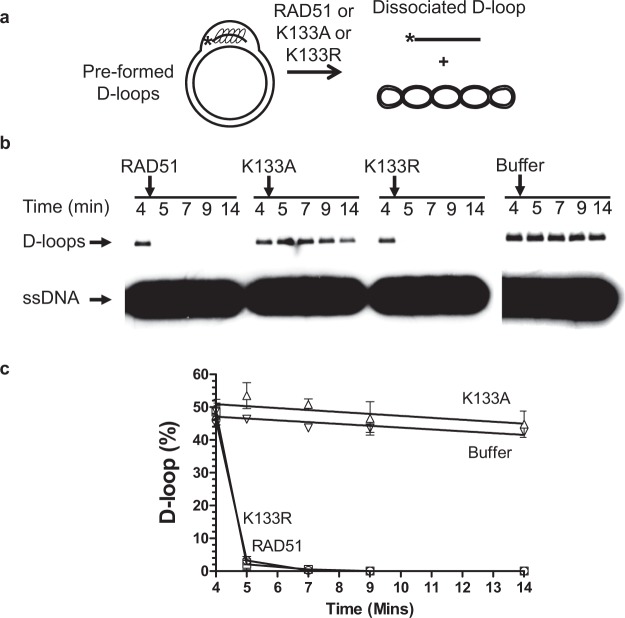
Effect of the RAD51 K133A and K133R mutations on D-loop dissociation. (**a**) Experimental scheme. (**b**) The kinetics of D-loop dissociation by RAD51, or the RAD51 K133A or K133R mutants. The addition of RAD51 (0.5 µM) or RAD51K133R (0.5 µM), but not RAD51K133A (0.5 µM), causes dissociation of RAD51-generated D-loops. As a control, the RAD51 storage buffer was used. D-loop dissociation was initiated at “4 min” time point since the beginning of D-loop formation. (**c**) Graphical representation of the data from panel (b). Error bars indicate SEM; the experiments were repeated at least three times.

To test this hypothesis, we used RAD51 mutant proteins, RAD51 K133R and RAD51 K133A. Both these proteins bind dsDNA, whereas only RAD51 K133R, but not RAD51 K133A, is proficient in dsDNA unwinding^19^ (Fig. S3b). We found that K133R, but not RAD51 K133A, promotes D-loop dissociation similar to wild type RAD51 (Fig. 2). Furthermore, when BRC4 was pre-incubated with wild type RAD51, BRC4 prevented the unwinding of dsDNA (Fig. S3c). Together, these data indicate that the DNA unwinding activity of RAD51 is required for D-loop dissociation.

### BRCA2 controls D-loop dissociation by RAD51

To promote DNA strand exchange *in vitro*, RAD51 needs to be present in a stoichiometric amount relative to ssDNA, which is 1 RAD51 monomer per 3 nt of ssDNA^20^. Previous work has demonstrated that the presence of excessive amounts of RAD51 can inhibit DNA strand exchange^21^. Binding of free excessive RAD51 to the donor dsDNA may prevent its productive interactions with the RAD51-ssDNA filament during the search for homology explaining the inhibitory effects. However, here we find that RAD51 may have a post-synaptic mechanism of action; actively dissociating D-loops, the product of DNA strand invasion, through DNA binding and unwinding activity. In early studies, it was shown that RecA, a bacterial homolog of RAD51, can also perform this action^18^.

BRCA2 is an important mediator protein that promotes RAD51 filament formation on ssDNA helping it to overcome inhibition by Replication Protein A (RPA), a ubiquitous ssDNA binding protein^4,5^. Previously, it was shown that BRCA2 directly binds RAD51 through BRC repeats and selectively targets RAD51 to ssDNA reducing non-productive interactions with dsDNA^4,5,7,22^. There are eight BRC repeats in human BRCA2 encoded by exon 11, with BRC4 having the greatest affinity for RAD51^8^. BRCA2 helps stabilize the RAD51-ssDNA filament by inhibiting RAD51 ATPase activity^4,5^. Here, we wanted to assess the ability of BRCA2 to counter D-loop dissociation by RAD51 through binding to this protein.

**Figure 3:**
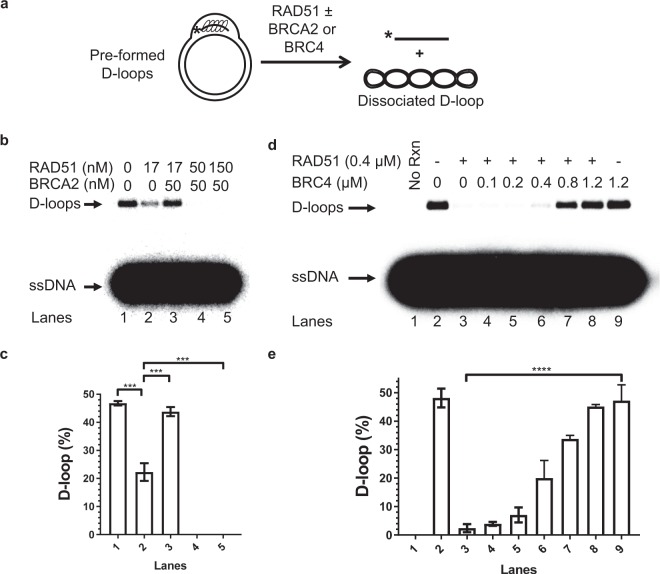
BRCA2 prevents D-loop dissociation by RAD51. (**a**) Experimental scheme of post-synaptic addition of BRCA2/BRC4 and RAD51. (**b**) Effect of BRCA2 on D-loop dissociation by RAD51. BRCA2 and RAD51 in the indicated concentrations were pre-incubated prior to addition to the pre-formed D-loops. (**c**) Data from (**b**) represented graphically. (**d**) Effect of BRC4 on D-loop dissociation by RAD51. BRC4 in the indicated concentrations was pre-incubated with RAD51 prior to addition to the pre-formed D-loops. Lane 1 (“No Rnx”) represents protein-free control. Lanes 2 and 9 represent D-loops not subjected to excess RAD51. (**e**) Graphical representation of data from (**d**). Error bars indicate SEM and the experiments were repeated at least three times. *p < 0.05, **p < 0.01, ***p < 0.001, ****p < 0.0001.

D-loops were formed by the addition of pUCFBR (0.25 µM, nt) to nucleoprotein filaments produced by RAD51 (40 nM) binding to ssDNA (0.12 µM, nt). The D-loops were then subjected to incubation with mixtures of BRCA2 and RAD51, in which the RAD51 concentration was varied (Fig. 3a). In the absence of BRCA2, RAD51 was able to dissociate D-loops under these conditions (Fig. 3b,c, lane 2). Furthermore, we found that BRCA2 can fully protect D-loops from dissociation by RAD51 (Fig. 3b,c, lane 3**)**. The strongest protective effect of BRCA2 was observed at the stoichiometry of 3 BRCA2 per 1 RAD51. By increasing the concentration of RAD51 the protective effect of BRCA2 was extinguished. This 3:1 stochiometric requirement for BRCA2 that carries 8 BRC repeat suggests that either not all BRCA2 molecules were active, or only the BRC repeat with the highest affinity for RAD51, like BRC4, was able to bind RAD51 under our experimental conditions.

To test whether the RAD51-BRC interaction was necessary and sufficient for the protective effect of BRCA2, we used the purified BRC4 peptide, as BRC4 showed the strongest interaction with RAD51 among all BRC repeats and is used as a proxy for BRCA2 when examining interactions with RAD51^4,5,22^. D-loops were formed by the incubation of pUCFBR (6.25 µM, nt) with RAD51-ssDNA filaments produced by RAD51 (1 µM) with ssDNA (3 µM, nt). We found that BRC4 alone can protect the D-loops from dissociation by RAD51 in a concentration dependent manner (Fig. 3d,e). A three-fold excess of BRC4 over RAD51 caused full D-loop protection; but even at a 2:1 BRC4:RAD51 ratio, BRC4 protected 70% of D-loops (Figs 3d,e and S4b,c). In contrast, RAD52, another HR protein that interacts with RAD51^23^, did not have any significant protective effect even at a 3-fold molar excess (Fig. S4b,c, lane 10). Furthermore, the effect of BRCA2 to control dissociation of RAD51-generated D-loops relies on specific RAD51-BRC interactions, as BRC4 had no effect on RAD54-mediated^12^ dissociation of RAD51-bound D-loops regardless of the presence or absence of EGTA that destabilizes the RAD51-DNA filament by chelating calcium ion (Fig. S5).

### BRC4 effect on RAD51 D-loop yield depends on RAD51 concentration

**Figure 4:**
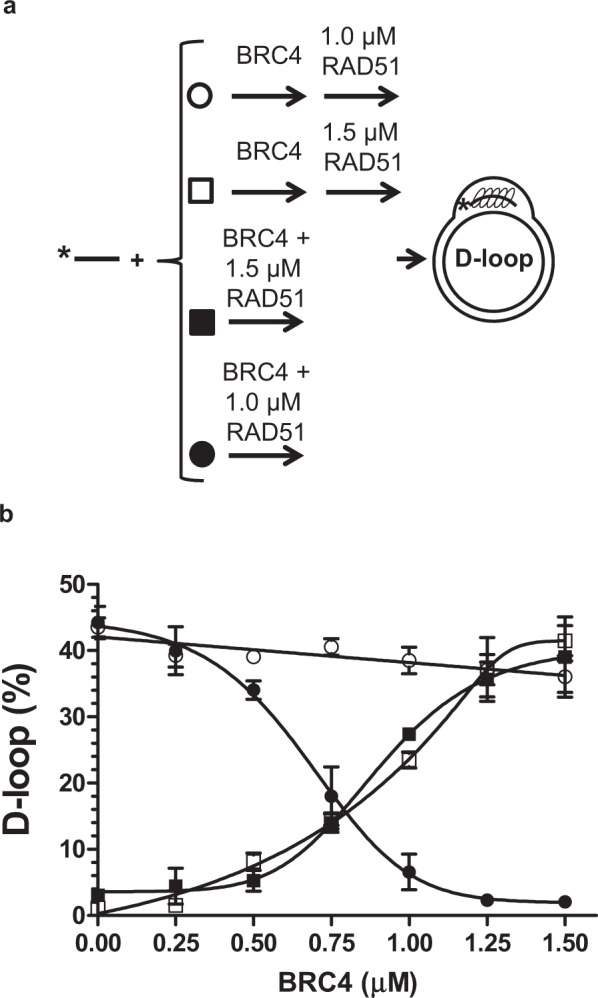
BRC4 has differential effects on D-loop yield depending upon initial RAD51 concentration. (**a**) Schematic depicting order of addition for BRC4 and RAD51 proteins to the ^32^P-ssDNA. BRC4 and RAD51 were either added sequentially (open symbols) or pre-incubated (closed symbols) before addition to the ^32^P- ssDNA. RAD51 was either at 1 µM (circles) or 1.5 µM (squares) concentration. (**b**) Effect of pre-synaptic combination of RAD51 and BRC4 on the D-loop yield. Representative gel images are shown in Fig. S7. Error bars indicate SEM and the experiments were repeated at least three times.

We then investigated whether BRC4 has a protective effect against D-loop dissociation by RAD51, when it is present during RAD51-ssDNA filament assembly (Fig. 4a), which likely mimics the conditions *in vivo*^24^. First, we determined that 1.5-fold excess of RAD51 (1.5 µM) over its optimal concentration (1 µM, or 1 RAD51 monomer per 3 nt of ssDNA) during filament assembly decreased the D-loop yield to nearly undetectable levels (Fig. S6). This decrease was consistent with D-loop dissociation by RAD51 unbound to ssDNA. At 1.5-fold RAD51 excess, BRC4, when added during filament assembly, rescued D-loop formation in a concentration-dependent manner (Figs 4b; and S7-right panels). The rescue effect of BRC4 was the same regardless of whether BRC4 was pre-incubated first with RAD51 (Fig. 4b, closed squares) or added to ssDNA together with RAD51 (Fig. 4b, open squares). These results are consistent with the ability of BRC4 to stimulate D-loop formation post-synaptically by removing the RAD51 excess. In contrast, when BRC4 was added to the reaction at the optimal RAD51 concentration (1 µM), there was nearly no effect on the D-loop yield (Figs 4b, open circles; S7a). Consistent with the previous observations^4^, this results suggests that the rate of RAD51 oligomerization is considerably faster than RAD51-BRC4 interaction. However, if BRC4 was pre-incubated with RAD51 (1 µM) prior to their addition to ssDNA, there was a precipitous drop in the D-loop yield (Figs 4b, closed circles; S7b) consistent with disruption of RAD51-ssDNA filament formation through binding of BRC4 to the RAD51 monomer-monomer interface^22^.

## Discussion

Our current results show that RAD51 can actively dissociate D-loop through unwinding of dsDNA. D-loop dissociation requires ATP binding by RAD51, but not ATP hydrolysis. We also show that BRCA2 may have a post-synaptic function in HR by modulating D-loop dissociation. Thus, BRCA2 can prevent D-loop dissociation by direct interaction with RAD51.

**Figure 5:**
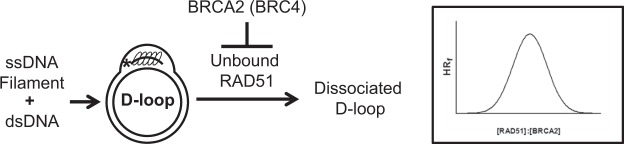
BRCA2 controls homologous recombination by regulating D-loop dissociation. An excess of RAD51 may cause D-loop dissociation, whereas BRCA2 modulates dissociation of D-loops via direct interaction with RAD51 through its BRC repeats. Inlet, the balance between RAD51 and BRCA2 at the site of DSB repair may determine the outcome of HR.

Our current results provide biochemical evidence that BRCA2-RAD51 homeostasis is crucial for HR control at the site of DNA damage (Fig. 5) as previously proposed by Baker and co-workers using cellular assays^16,17^. As D-loop dissociation is the delineation between the Synthesis Dependent Strand Annealing (SDSA) and the Double-Strand Break Repair (DSBR) pathway, this fine-tuning mechanism based on the RAD51/BRCA2 balance may also contribute to the choice between these two pathways to control cross-overs that are generated in DSBR pathway, thereby maintaining genomic integrity^25,26^. While there are multiple helicases^11–15,27,28^ that may dissociate D-loops, they all require additional spatiotemporal recruitment which is not needed for RAD51/BRCA2 because they are already present at the DSB repair site.

Several reports indicate that the balance between BRCA2 and RAD51 is an important factor in HR. RAD51 expression was found elevated in a wide range of tumors^29^, which is thought to increase the DNA repair efficiency by HR causing higher resistance to chemo- and radiation therapy^30^. However, RAD51 overexpression in cells has led to conflicting reports showing either decrease^16,31,32^ or increase^17,30,33,34^ in HR efficiency. Our current results provide a rationale for this apparent controversy. An increase in RAD51 concentration without the corresponding increase of BRCA2 results in dissociation of D-loops by RAD51, whereas an excess of BRCA2/BRC4 inhibits RAD51 filament formation and the D-loop yield, e.g., when BRC4 was pre-incubated with the optimal RAD51 (1 µM) concentration prior to addition to ssDNA (Figs 4b and S7). In both cases, it is expected that HR would be inhibited *in vivo* (Fig. 5, inset). In contrast, we expect that an increase in BRCA2 expression concomitant with the RAD51 increase, would neutralize the dissociative effect of RAD51 leading to an overall increase of HR^4,5^. These predictions from our *in vitro* results are consistent with available cellular data. An increase in HR was observed when both RAD51 and BRCA2 expression levels were increased^32^; whereas a decrease in HR was observed when RAD51 was overexpressed with no change in BRCA2 expression^17^, or when BRCA2/BRC4 was overexpressed with no change in RAD51 levels^16,35^.

Previous work from the Jasin lab demonstrated an inverse relationship between canonical HR and the error-prone Single-Strand Annealing (SSA) pathway^36^. When RAD51 or BRCA2 was impaired, the HR was reduced but SSA was increased. Taken together, these data and our current biochemical results suggest that shifting the optimal RAD51/BRCA2 balance may not only decrease the HR but cause an increase in SSA.

Lastly, our findings also apply to HR-based genome editing tools such as CRISPR systems, as one of the limitations for this technology is the low incidence of HR. While current approaches are focused on diversion of DSB repair away from Non-Homologous-End-Joining (NHEJ) and towards HR^37,38^, it would be attractive to complement these approaches by increasing overall HR efficiency. Our results imply that this can be achieved by coordinated overexpression of key HR proteins, like RAD51 and BRCA2.

## Methods

### Proteins and DNA

Human RAD51, RAD51 K133A, RAD51 K133R^39^, RAD54^40^, BRC4^4^, BRCA2^5^ were purified as described previously. [γ-^32^P] ATP was purchased from PerkinElmer Life Sciences. The oligonucleotide sequences used in the study are presented in Table S1. The oligonucleotides (IDT.Inc.), pUC19, pUCFBR were purified as described previously^39^. 3′-tailed dsDNA substrate was prepared by annealing of equimolar (molecules) amounts of complementary oligonucleotides #209 and #199. pUCFBR DNA was constructed by inserting 84 bp (Sall/HindIII) fragment from φX174 and 658 bp (EcoRI/Sall) fragment from pBR327 into pUC19 (Fig. S8). Unless indicated otherwise, the DNA concentrations are expressed as moles of nucleotide.

### D-loop formation

To form the nucleoprotein filament, ^32^P-labeled ssDNA (3 µM, nt #160) was incubated with RAD51 (1 µM) in buffer containing 25 mM Tris-acetate, pH 7.5, 20 mM KCl, 1 mM DTT, 1 mM ATP, 100 µg ml^−1^ BSA, and 2 mM CaCl_2_. As we showed previously, Ca^2+^ increases the efficiency of D-loop formation by RAD51 and reduces dissociation of RAD51 from DNA^39^. Unless otherwise indicated, the mixture was incubated for 15 min at 37 °C. To form D-loops, homologous pUCFBR (6.25 µM, nt) scDNA was added to the mixture and incubated at 37 °C for the indicated period. When D-loops were produced using 3′-tailed dsDNA (#209/199, 30 nM, molecules or 3 µM nt/bp), the reaction was initiated by addition of homologous pUC19 scDNA (50 µM, nt). All reactions were terminated by addition of 1% SDS and 880 µg ml^−1^ proteinase K, followed by incubation for a minimum of 10 min at 37 °C. 0.1 volume of loading buffer (70% glycerol and 0.1% bromophenol blue) was added, and the samples were analyzed by electrophoresis (10 V cm^−1^ for 1.5 h) in a 1% agarose gel in TAE buffer (40 mM Tris acetate, pH 8.0 and 1 mM EDTA). Gels were dried on DEAE paper, the ^32^P-radioactivity bands were visualized by Typhoon FLA 7000 and quantified by ImageQuant TL. The D-loop yield is expressed as a percentage of plasmid DNA molecules carrying D-loops relative to the total plasmid DNA. It was calculated using the formula: [X/(X + Y)*100%]*Z. Where, X is the amount of ^32^P radioactivity in the ssDNA that was incorporated into D-loops; Y – is the amount of ^32^P radioactivity in free ssDNA; Z is the ratio of the molar molecule concentrations of ssDNA/plasmid DNA.

When indicated, the agarose gels were stained with Ethidium Bromide (EtBr) (2 µg ml^−1^) by incubation for 1 h followed by destaining with nanopure water for 1 h. The DNA bands were visualized using an AlphaImager 3400 system. Auto-contrast and/or inversion were applied using Image Ready 7.0 (Adobe).

### D-loop dissociation with RAD51, the RAD51 mutants, or RAD54

To dissociate D-loops with RAD51, RAD51 K133A, RAD51 K133R, the proteins were directly added to pre-formed D-loops in the D-loop formation buffer. The reactions were carried out at 37 °C. When indicated, RAD51 was first incubated with BRCA2, BRC4, RAD52 or pUC19 DNA at the indicated concentrations for 15 min on ice in protein dilution buffer containing BSA (100 µg ml^−1^) prior to addition to the D-loops. To dissociate D-loops with RAD54, ATP regeneration system was added (0.02 M PhosphoCreatine with 0.03 U Phosphocreatine Kinase) to the reaction mixture along with magnesium acetate (1 mM). Before adding RAD54 (50 nM), the temperature of the reaction mixture was reduced from 37 °C to 30 °C. The D-loop dissociation reactions proceeded for 10 min (unless otherwise indicated) and the products were analyzed as described in D-loop formation section.

### DNA Unwinding Assay

Relaxed DNA was prepared in a 50 µl reaction mixture by incubating negatively supercoiled plasmid pUC19 DNA (0.5 µg) with calf thymus Topoisomerase I (1 Unit; Invitrogen) in buffer containing 50 mM Tris HCl pH 7.5, 50 mM KCl, 10 mM MgCl_2_, 0.5 mM DTT, 0.1 mM EDTA, 100 µg ml^−1^ BSA for 30 min at 37 °C. To test the unwinding activity of RAD51, RAD51 K133A, RAD51 K133R, these proteins were incubated with relaxed pUC19 (25 µM, nt) and unlabeled #160 (12.5 µM, nt) in the D-loop formation buffer supplemented with 10 mM magnesium acetate^19^ for 5 min at 37 °C. When BRC4 was used, the proteins were preincubated with BRC4 on ice for 15 min prior to addition to the reaction. Then, 1 U of calf thymus Topoisomerase I was added followed by incubation for 20 min at 37 °C. The reaction was terminated as described in D-loop formation section. The samples were analyzed by electrophoresis on 1.5% agarose gel containing TAE (40 mM Tris acetate, pH 8.0, and 1 mM EDTA) at 20 V cm^−1^ for 1.5 h. The gels were stained with Ethidium Bromide (2 µg ml^−1^) for 1 h and destained with nanopure water for 24 h. The DNA bands were visualized using an AlphaImager 3400 system, and auto-contrast was applied using Image Ready 7.0 (Adobe).

### D-loop deproteinization

D-loops formed, as described in D-loop formation section, were then deproteinized by addition of 1% SDS and 880 µg ml^−1^ proteinase K, followed by incubation for 20 min at 37 °C. The deproteinized D-loops were then purified twice by size exclusion chromatography on S-400 spin-columns (GE Healthcare) equilibrated with 25 mM Tris-acetate (pH 7.5) buffer. The purified D-loops were then mixed into the D-loop formation buffer and used as substrates for RAD51-promoted D-loop dissociation reaction by incubating with RAD51 at indicated concentrations. The reactions were terminated and analyzed as described in D-loop formation section.

### Statistical analysis

All statistical analyses were conducted in Prism 5.0 (GraphPad). Significance between groups was calculated using ordinary one-way ANOVA. Statistical significance throughout paper is represented with ns = p > 0.05, *p < 0.05, **p < 0.01, ***p < 0.001.

## Supplementary information

Supplementary Information

## Data Availability

The data supporting the findings of this study within the paper and its Supplementary Information files are available from the corresponding author upon request.
