# Supplementary material for: The Post-Synaptic Function of Brca2
Source: Sci Rep. 2019 Mar 14;9:4554. doi: 10.1038/s41598-019-41054-y (PMC6418147; doi:10.1038/s41598-019-41054-y)
Supplement: Supplementary file 1 — Supplementary Information [file 41598_2019_41054_MOESM1_ESM.pdf]

THE POST-SYNAPTIC FUNCTION OF BRCA2

Supplementary Information

Charles X. Wang[1,2,#], Judit Jimenez-Sainz[3], Ryan B. Jensen[3], Alexander V. Mazin[1*]

[1]Department of Biochemistry and Molecular Biology, Drexel University College of Medicine, Philadelphia, PA 19102;
[2]MD/PhD Program, Drexel University College of Medicine, Philadelphia, PA 19102;
[3]Department of Therapeutic Radiology, Yale University School of Medicine, New Haven, CT 06520, USA;
[#]Present Address: Department of Radiation Oncology, MD Anderson Cancer Center, Houston, TX 77030

Content:

Figures S1-S7

Table S1

Figure S1

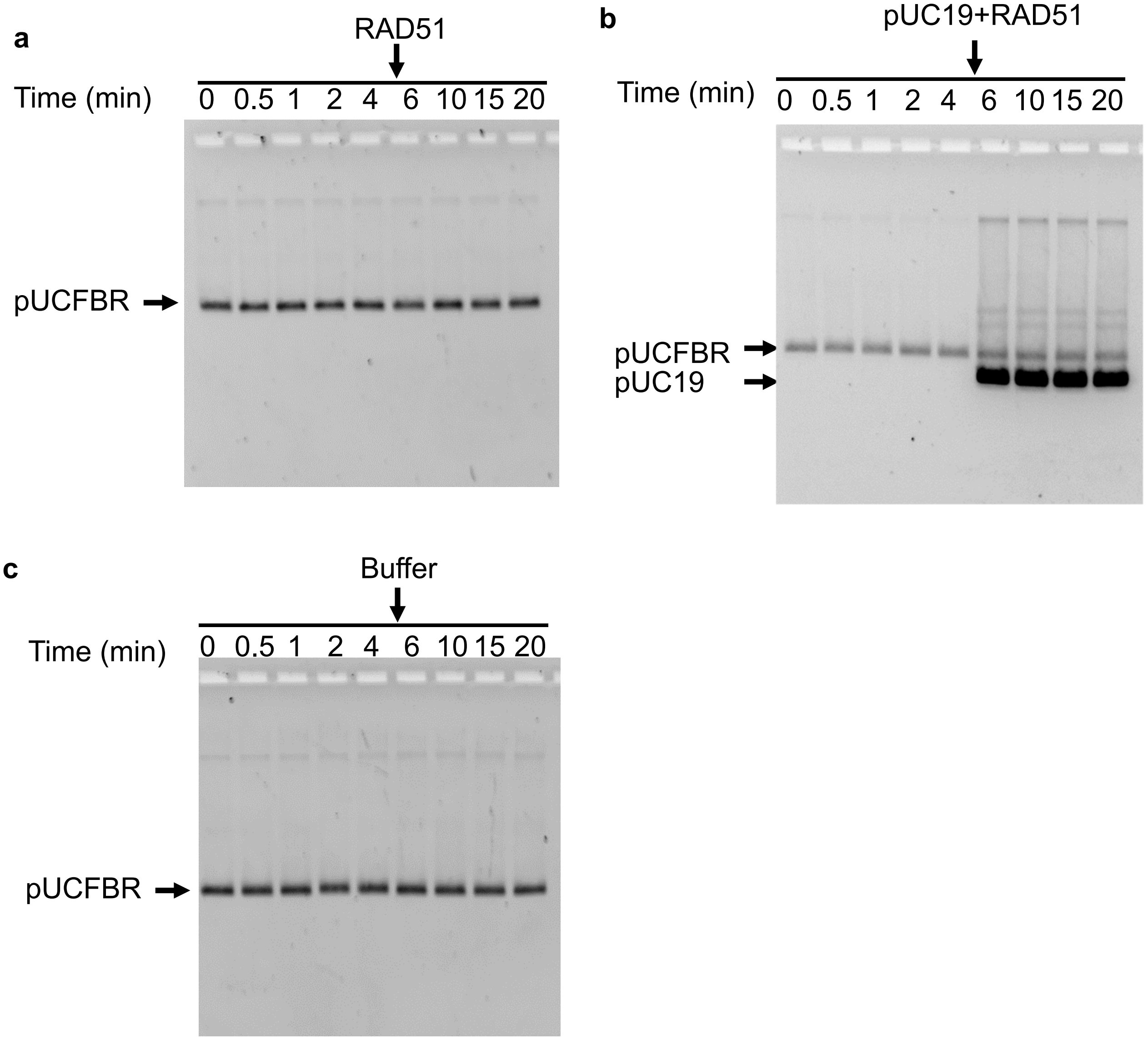
a
RAD51
Time (min)
0 0.5 1 2 4 6 10 15 20
pUCFBR
b
pUC19+RAD51
Time (min)
0 0.5 1 2 4 6 10 15 20
pUCFBR
pUC19
c
Buffer
Time (min)
0 0.5 1 2 4 6 10 15 20
pUCFBR

Figure S1. Dissociation of D-loops is not due to reaction buffer contaminants or nicking of supercoiled plasmid DNA.

Agarose gels shown in **Fig. 1** after autoradiography were stained with Ethidium Bromide (EtBr) to visualize DNA bands. No conversion of scDNA into an open-circle form was observed after addition of (a) RAD51, (b) pUC19+RAD51, (c) reaction buffer. The experiments were repeated at least three times.

Figure S2

a

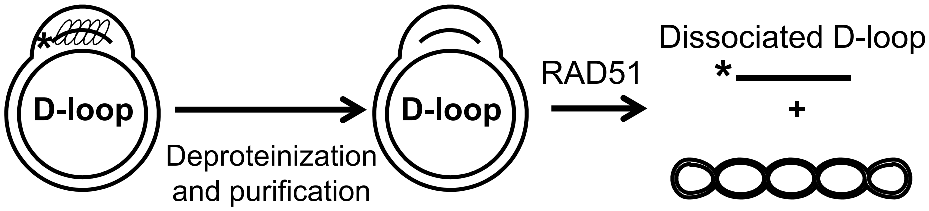

b

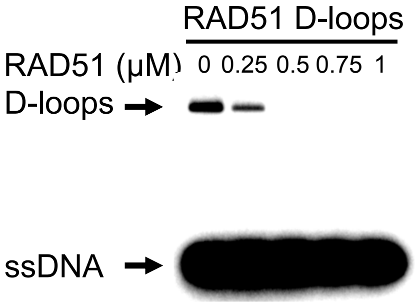

c

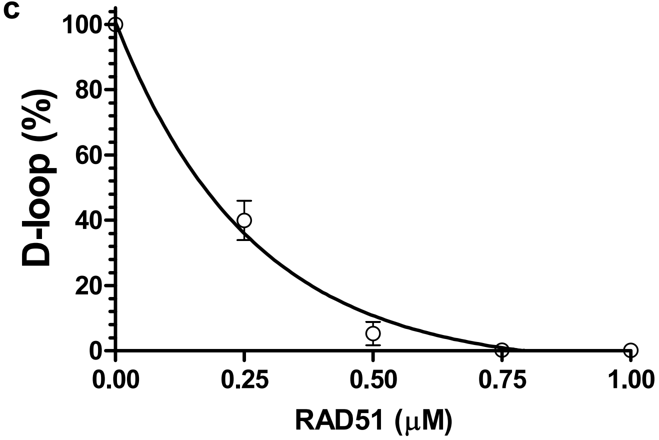

Figure S2. RAD51 efficiently dissociates deproteinized D-loops.

(a) Experimental scheme: RAD51-generated D-loops were deproteinized, then purified by size exclusion chromatography (S-400) twice, prior to addition of RAD51. (b) of D-loop dissociation by RAD51 was analyzed by electrophoresis in a 1% agarose gel. (c) Normalized D-loop yields from (b) the actual maximum yield was 47% for RAD51-generated D-loops. Error bars indicate SEM; experiments were repeated at least three times.

a **Figure S3**

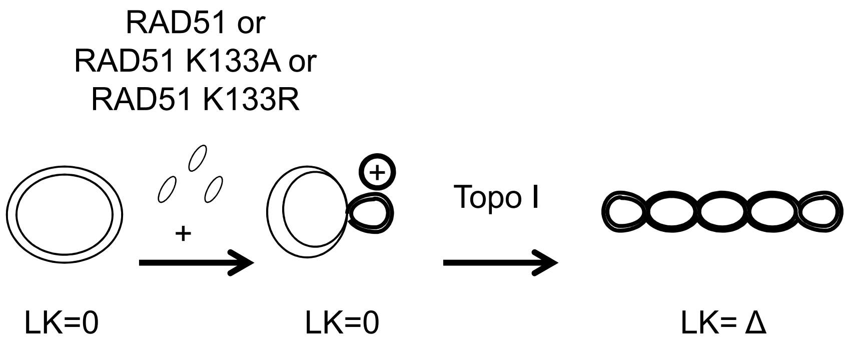

b

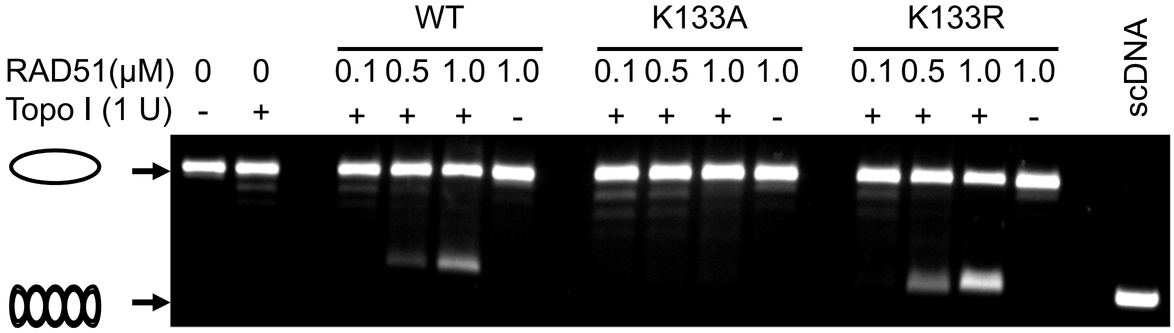

c

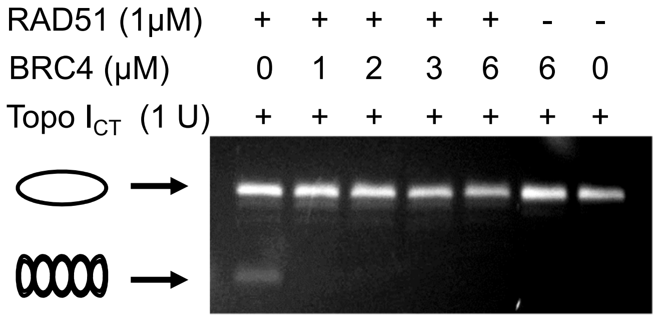

Figure S3. DNA unwinding activity of RAD51, RAD51 K133A, and RAD51 K133R.

(a) Experimental scheme. Relaxed pUC19 DNA (25 μM, nt) was incubated with RAD51, RAD51 K133A, or RAD51 K133R at the indicated concentrations. Accumulated topological stress due to dsDNA unwinding by RAD51 or the RAD51 mutants is released by calf thymus Topoisomerase I (1 U) leading to a change of linker number (LK) which can then be detected by a change in mobility during electrophoresis in agarose gels. (b) The DNA unwinding activity of RAD51, RAD51 K133R, and RAD51 K133A visualized by electrophoresis in a 1.5% agarose gel. (c) Effect of BRC4 on RAD51 unwinding activity. BRC4 and RAD51 were pre-incubated prior to addition to DNA. All experiments were repeated at least three times.

Figure S4

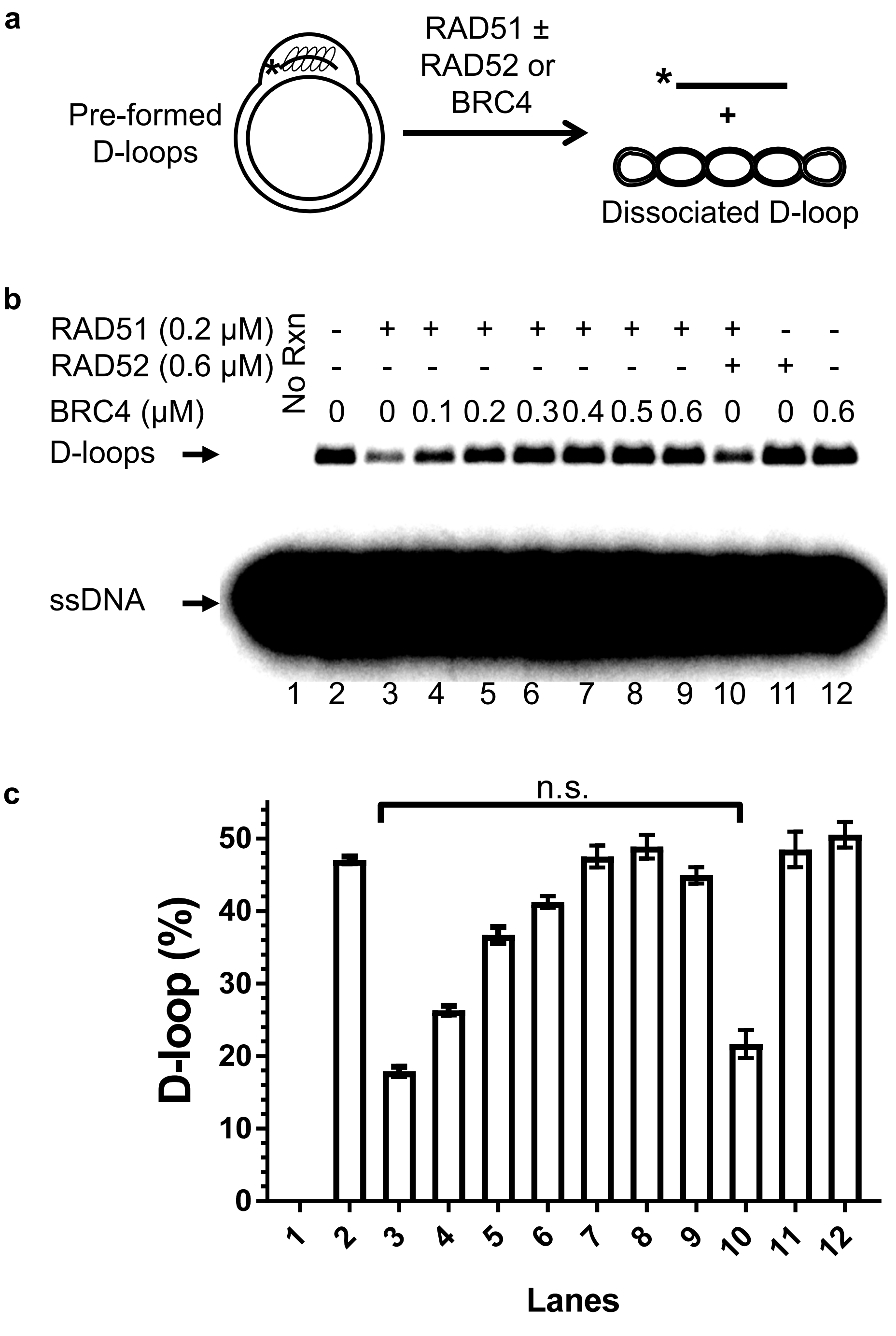
a
Pre-formed D-loops
RAD51 ± RAD52 or BRC4
Dissociated D-loop
b
RAD51 (0.2 μM) - + + + + + + + + - -
RAD52 (0.6 μM) - - - - - - - - + + -
BRC4 (μM) 0 0 0.1 0.2 0.3 0.4 0.5 0.6 0 0 0.6
No Rxn
D-loops
ssDNA
1 2 3 4 5 6 7 8 9 10 11 12
c
n.s.
D-loop (%)
0
10
20
30
40
50
1 2 3 4 5 6 7 8 9 10 11 12
Lanes

Figure S4. Effect of BRC4 and RAD52 on RAD51-mediated D-loop dissociation.

(a) Experimental scheme. (b) Pre-incubated mixture of BRC4+RAD51 or RAD52+RAD51 was added to the D-loops. (c) Data from (b) represented graphically. Error bars indicate SEM; experiments were repeated at least three times.

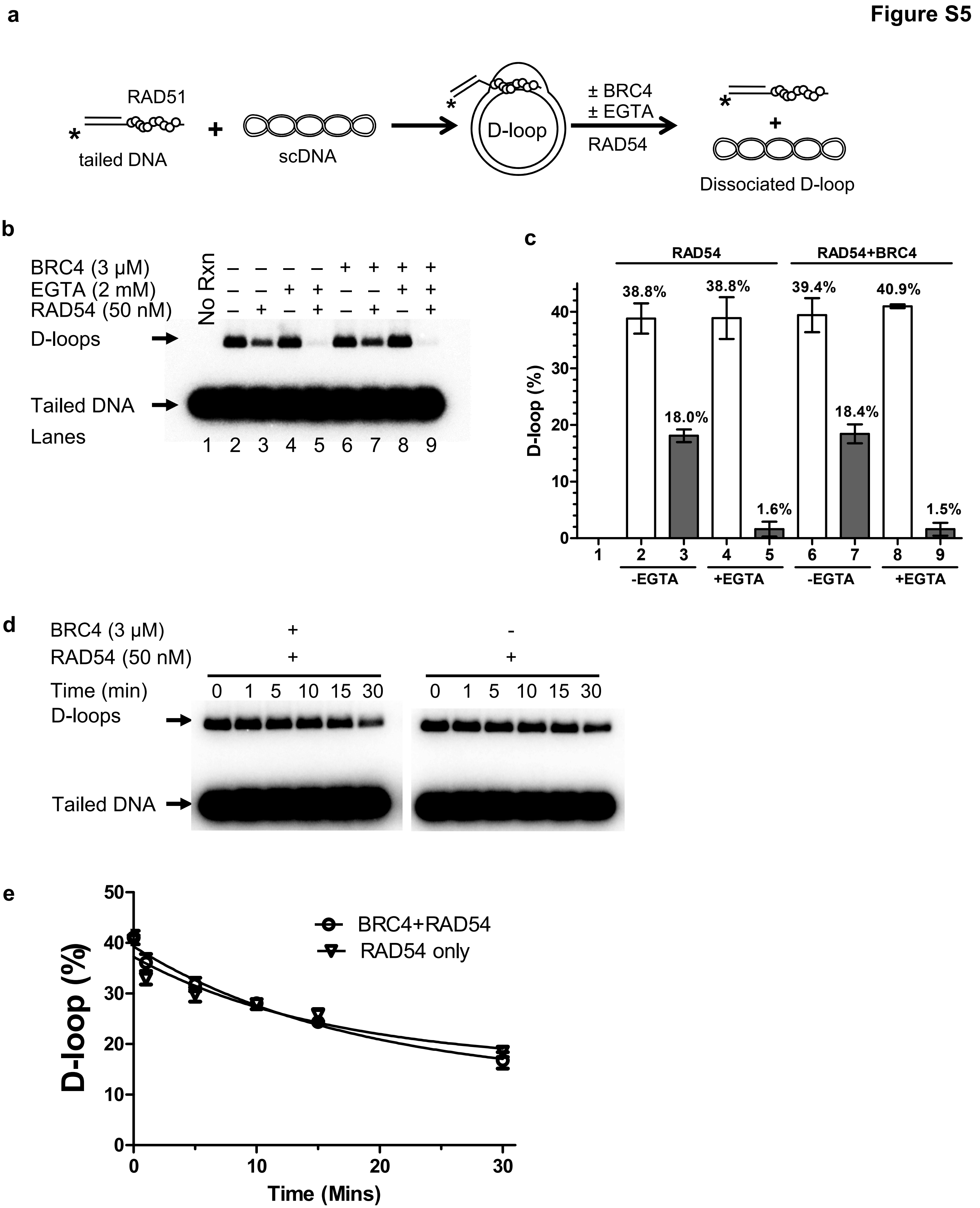
a
RAD51
tailed DNA
scDNA
D-loop
± BRC4
± EGTA
RAD54
Dissociated D-loop
b
BRC4 (3 μM)
EGTA (2 mM)
RAD54 (50 nM)
No Rxn
D-loops
Tailed DNA
Lanes
1 2 3 4 5 6 7 8 9
c
RAD54
RAD54+BRC4
D-loop (%)
38.8%
18.0%
38.8%
1.6%
39.4%
18.4%
40.9%
1.5%
-EGTA
+EGTA
-EGTA
+EGTA
d
BRC4 (3 μM)
RAD54 (50 nM)
Time (min)
0 1 5 10 15 30
D-loops
Tailed DNA
e
BRC4+RAD54
RAD54 only
D-loop (%)
Time (Mins)

Figure S5. BRC4 has no effect on RAD54-mediated D-loop dissociation.

(a) Experimental scheme. RAD51 (1 μM) was incubated with 3'-tailed dsDNA (3 μM nt/bp), and D-loop formation was initiated by the addition of pUC19 (50 μM, nt). The D-loops were subjected to dissociation by RAD54 in the presence or absence of BRC4 or EGTA for 30 min at 30 °C. Lane 1, "No Rxn" denotes protein-free control. (b) Effect of BRC4 on RAD54 mediated D-loop dissociation. (c) Data from (b) plotted as a graph. (d) The kinetics of D-loop dissociation by RAD54 in the presence or absence of BRC4. (e) Graphical representation of (c). Error bars indicate SEM and the experiments were repeated at least three times.

Figure S6

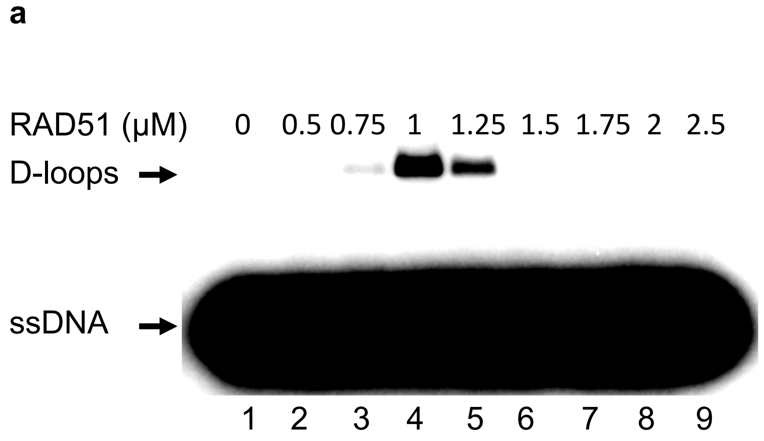

a
RAD51 (μM) 0 0.5 0.75 1 1.25 1.5 1.75 2 2.5
D-loops
ssDNA
1 2 3 4 5 6 7 8 9

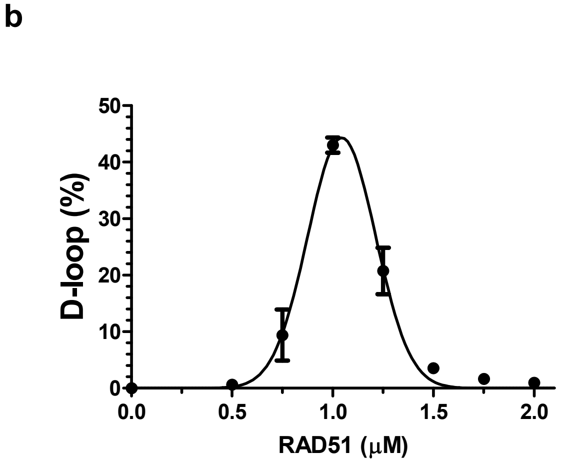

b
D-loop (%)
50
40
30
20
10
0
0.0 0.5 1.0 1.5 2.0
RAD51 (μM)

Figure S6. Effect of RAD51 concentration on the D-loop yield (a) Visualization of D-loop formation by electrophoresis in a 1% agarose gel. To form nucleoprotein filament, RAD51 at the indicated concentrations was incubated with ssDNA (3 μM, nt). D-loop formation was initiated by addition of pUCFBR (6.25 μM, nt). (b) Data in (a) plotted as a graph. Error bars indicate SEM; experiments were repeated at least three times.

Figure S7

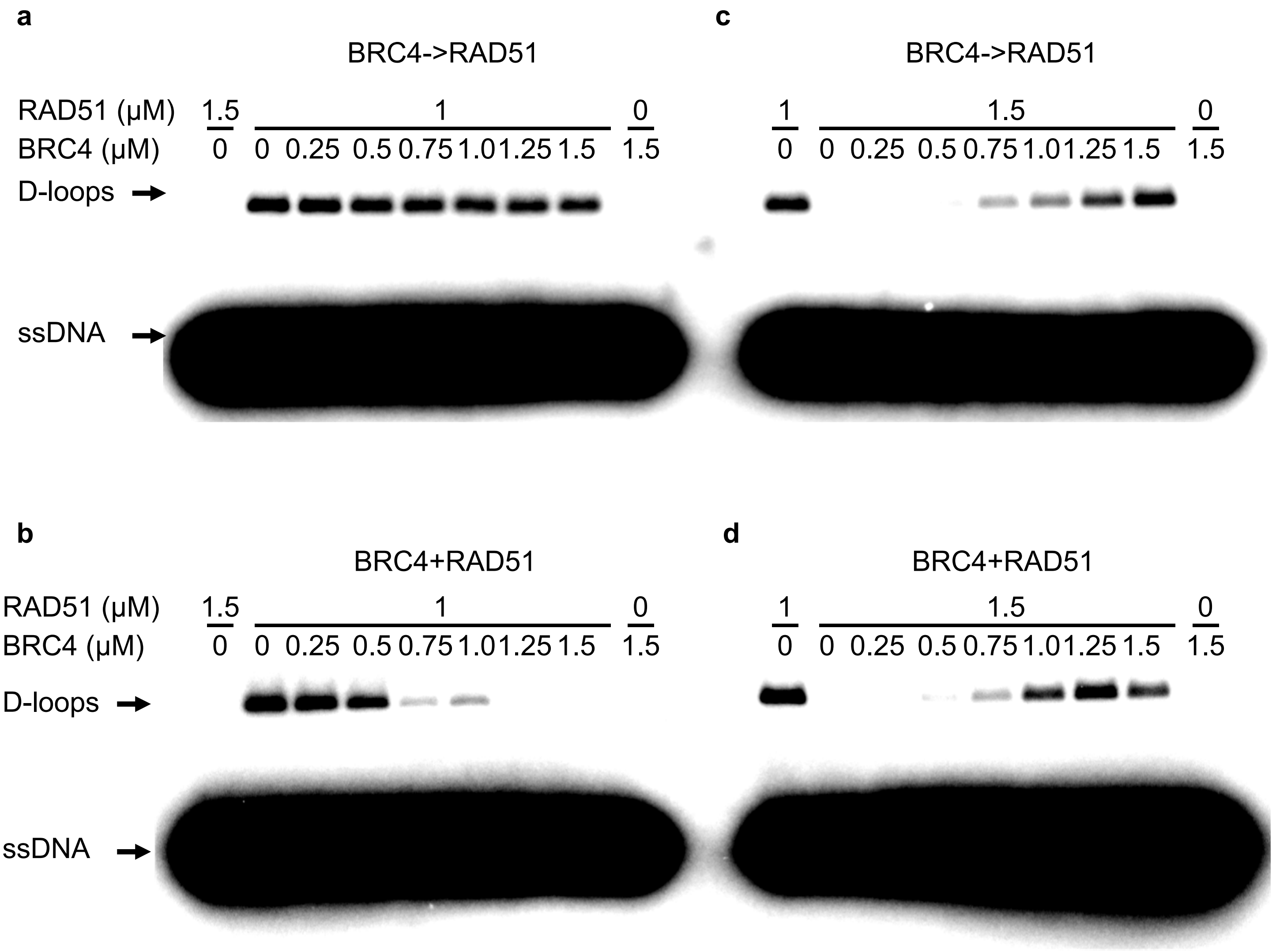
a
BRC4->RAD51
RAD51 (μM) 1.5 1 0
BRC4 (μM) 0 0 0.25 0.5 0.75 1.0 1.25 1.5 1.5
D-loops
ssDNA
c
BRC4->RAD51
1 1.5 0
0 0 0.25 0.5 0.75 1.0 1.25 1.5 1.5
b
BRC4+RAD51
RAD51 (μM) 1.5 1 0
BRC4 (μM) 0 0 0.25 0.5 0.75 1.0 1.25 1.5 1.5
D-loops
ssDNA
d
BRC4+RAD51
1 1.5 0
0 0 0.25 0.5 0.75 1.0 1.25 1.5 1.5

Figure S7. Effect of order of addition of RAD51 and BRC4 during RAD51-ssDNA filament formation on the D-loop yield. RAD51 was either at the optimum concentration (1 μM; a and b) or in excess (1.5 μM; c and d). RAD51 and BRC4 were either preincubated prior to addition to the reaction (b and d) or added sequentially: BRC4 then RAD51 (a and c). Graphical representation of these data is shown in **Fig. 3g**.

Table S1. List of DNA oligonucleotides used in this study.

Oligo ID #	Length (nt)	5'→3' Sequence	Feature
#160	84	AGCTTTGAGATGCTTGCTTATC AACAGAAGGAGTTACCTCGCG TTGCGTCTATTATGGTACCACA CCAATCTTTCCAAGCAACG	Full Homology to pUCFBR
#209	100	**AAT TCT CAT TTT ACT TAC CGG ACG CTA TTA GCA G**TG GGT GAG CAA AAA CAG GAA GGC AAA ATG CCG CAA AAA AGG GAA TAA GGG CGA CAC GGA AAT GTT G	64 nt Homology to pUC19
#199	36	CACTGCTAATAGCGTCCGGTAA GTAAAATGAGAATT	Full complement to #290

In nucleotide sequence of #209 a region of heterology to pUC19 is indicated by bold font.
